# The Effect of Dietary Metabolisable Energy and Stocking Density on Growth Performance, Blood Metabolites, Nutrient Utilization, and Carcass Traits in Broiler Chicks

**DOI:** 10.1002/fsn3.70836

**Published:** 2025-09-01

**Authors:** Zeyad Kamal Imari, Saeid Jahandost Ardin, Ahmad Hassanabadi

**Affiliations:** ^1^ Department of Animal Production Techniques, Technical College of Al‐Musaib Al‐Furat Al‐Awsat Technical University AL‐Kufa Iraq; ^2^ Department of Animal Science, Faculty of Agriculture Ferdowsi University of Mashhad Mashhad Iran

**Keywords:** broiler, digestibility, energy, performance, stocking density

## Abstract

This study aimed to evaluate the effects of dietary metabolisable energy (ME) levels and stocking density (SD) on growth performance, carcass traits, blood metabolites, and apparent total tract digestibility (ATTD) of nutrients over a 42‐day feeding trial in broiler chicks. A total of 468 one‐day‐old male Ross 308 broiler chicks were used in a completely randomized design featuring a 3 × 3 factorial arrangement. The experiment included three SDs (10, 13, and 16 birds/m^2^; SD10, SD13 and SD16, respectively) and three dietary ME levels: 3% lower than the recommendation, recommended for the strain, and 3% higher than the recommendation (recommended‐energy, high‐energy, and low‐energy diets, respectively), with five replicates per treatment. During the starter phase, feed intake (FI) was significantly (*p* < 0.05) lower in broilers fed high‐energy diets compared to those fed low‐energy and recommended diets. Additionally, SD16 exhibited higher FI than SD10 and SD13 (*p* < 0.05). Birds fed recommended and high‐energy diets showed greater weight gain (WG) during the finisher and overall experimental periods compared to those fed low‐energy diets. WG in SD16 was significantly higher than in SD10 during the starter phase (*p* < 0.05). The feed conversion ratio (FCR) was significantly increased in birds fed low‐energy diets compared to the other two groups. Blood cholesterol, triglycerides, and HDL‐C concentrations were significantly higher in the recommended and high‐energy diet groups compared with the low‐energy group (*p* < 0.05). ATTD for lipids was notably lower in chicks fed low‐energy diets than in those on the other two diets (*p* < 0.05). Overall, high and recommended energy levels in the diet increased WG and improved FCR, but elevated blood cholesterol concentration. Increasing the stocking density to 16 birds/m^2^ did not negatively impact broilers performance.

## Graphical abstract

High energy levels in the diet increased weight gain and improved feed conversion ratio. Increasing the stocking density to 16 birds/m^2^ did not negatively impact broilers performance.

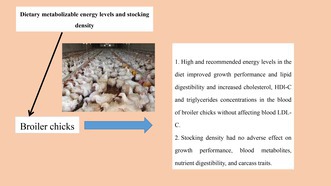

## 1 Introduction

The stocking density of broilers is a critical factor influencing the welfare and productivity of commercial poultry operations. Defined as the number of birds per unit area or the space allocated per bird, SD significantly impacts various economic aspects of the poultry industry. The globally accepted standards suggest achieving a body weight between 30 and 38 kg/m^2^ or maintaining a density of 20 birds/m^2^ by 35 days of age (Weeks and Butterworth 2004). However, broiler breeders often choose higher SDs to enhance profitability per unit area (Tsiouris et al. 2015), despite the potential detrimental effects on bird performance (Vanhonacker et al. 2009).

It has been demonstrated that elevated SD adversely affects growth and slaughter weight by 42 days of age (Simitzis et al. 2012). Uzum and Toplu (2013) reported that high SD (18 and 20 birds/m^2^) negatively impacted FI, growth rate, and FCR in broilers from 21 days of age onwards. Abudabos, Samara, Hussein, Al‐Atiyat, et al. (2013) showed that even a short period of high stocking density for only 5 days increases stress in broiler chickens and compromises their welfare. The consequences of high SD include increased ammonia levels in housing, litter humidity, prevalence of coccidiosis, higher incidence of foot pad lesions, thermal stress, and reduced mobility (Singh et al. 2021). Furthermore, Nahashon et al. (2009) found that high SD negatively affects overall carcass performance, while Simitzis et al. (2012) noted a significant reduction in muscle fat associated with high SD (27.2 kg/m^2^). Consequently, exceeding optimal SD adversely impacts both the welfare and profitability of broilers, while suboptimal SD can lead to inefficient space utilization and reduced profitability.

High SD can also lead to diminished body weight (Davami et al. 1987) and abnormal skeletal growth in modern broilers, primarily characterized by increased tibial curvature and reduced fracture strength (Buijs et al. 2012). Excessive bone curvature may result in lameness, thereby compromising bird welfare. Proper mineralization of bones relies on adequate dietary calcium and phosphorus, which are absorbed through the intestine and kidney (Bar et al. 1990). The relationship between SD and bone mineralization has been documented (Sun et al. 2013), with high SD negatively impacting bone ash and phosphorus content. However, there is limited research on the effects of SD on nutrient absorption.

The efficiency of digestion and nutrient absorption in broilers directly influences growth performance and overall health. Both feed formulation and environmental conditions, including SD, significantly affect nutrient digestibility. Rahbari et al. (2025) reported that broilers subjected to high SD exhibited reduced digestibility of apparent Metabolizable energy and crude protein (CP).

High SD is recognized as a stressor, with limited space linked to endocrine and behavioral changes indicative of stress, ultimately reducing welfare (Swanson 1995). Stress activates the hypothalamic–pituitary–adrenal axis, which plays a vital role in coordinating physiological and immunological responses (Dohms and Metz 1991). Blood biochemical profiles serve as indicators of the physiological and metabolic status of broilers (Zhang et al. 2018). High SD has been associated with metabolic alterations in blood parameters, including decreased lymphocyte counts, increased heterophil levels, and elevated heterophil‐to‐lymphocyte ratios (Astaneh et al. 2018). Additional metabolic changes include increased blood stress hormones (Najafi et al. 2015), diminished immune response, heightened oxidative stress (Gursu et al. 2004), and increased plasma glucose, corticosterone, and cholesterol levels (Shakeri et al. 2014).

Moreover, dietary ME levels significantly influence the intake and utilization of other nutrients. Energy intake is well‐documented to affect body composition in broilers (Wiseman and Lewis 1998).

As broiler chickens' SD increases, their access to feed decreases. We hypothesized that by increasing the level of ME and dietary nutrients, they may obtain their energy and nutrient requirements. Therefore, this study aimed to evaluate the effects of different SDs, combined with varying ME and nutrient levels in the diet, on the growth performance, nutrient digestibility, blood metabolites, and carcass traits in broiler chicks.

## 2 Materials and Methods

### 2.1 Experimental Design

All experimental procedures were conducted in strict accordance with ethical guidelines for animal experimentation and care, as mandated by the local ethics committee and the Animal Care and Use Committee of the Ferdowsi University of Mashhad. A total of 468 one‐day‐old male commercial Ross 308 broiler chicks, with a similar initial body weight of 40 ± 2 g, were used in a completely randomized design structured as a 3 × 3 factorial arrangement, resulting in 9 treatments with 5 replicates per treatment. Each replicate housed either 10, 13, or 16 birds/m^2^, for a 42‐day feeding trial conducted in floor pens measuring 1.0 m × 1.0 m within an environmentally controlled room. Inside each floor pen, there was a bell feeder with an area of 900 cm^2^ and two nipple drinkers.

The SDs employed were categorized into three levels: SD10 (10 birds/m^2^), SD13 (13 birds/m^2^), and SD16 (16 birds/m^2^). Additionally, three dietary levels of ME were applied: low‐energy (3% less than the recommended level), recommended‐energy (as per the guidelines for the Ross 308 strain, 2019), and high‐energy (3% more than the recommended level). The diets were formulated to maintain consistent ratios of ME to CP and ME to all other nutrients across each breeding phase. The experimental diets and their chemical compositions are detailed in Table 1 (Aviagen 2019). All birds had free access to a mash diet and water throughout the experiment. The environmental conditions included a temperature‐controlled room with a 23 L: 1D lighting program. The room temperature was maintained at 32°C–34°C for the first 3 days, gradually decreasing by 0.5°C weekly to a final temperature of 21°C.

**TABLE 1:** Ingredients and nutrient composition of experimental diets, *as‐fed basis*.

	Starter phase (1–10 day)			Grower phase (11–24 day)			Finisher phase (25–42 day)		
				ME level^a^					
Ingredients (%)	Low	Rec	High	Low	Rec	High	Low	Rec	High
Corn	58.04	52.68	47.72	62.31	58.14	53.22	64.66	60.23	57.12
Soybean meal (44% CP)	34.59	37.53	40.01	27.3	28.99	31.48	28.89	30.86	31.82
Fish meal	2.0	2.0	2.0	5.0	5.0	5.0	—	—	0.5
Soy Oil	1.47	3.83	6.18	2.25	4.57	6.89	3.12	5.32	6.76
Dicalcium phosphate	0.9	0.9	0.94	1.0	1.13	1.2	0.9	0.93	0.91
Calcium carbonate	1.53	1.6	1.65	0.95	0.97	0.99	1.39	1.43	1.46
Vitamin premix^b^	0.25	0.25	0.25	0.25	0.25	0.25	0.25	0.25	0.25
Mineral premix^c^	0.25	0.25	0.25	0.25	0.25	0.25	0.25	0.25	0.25
DL‐Methionine	0.35	0.36	0.39	0.26	0.28	0.3	0.1	0.25	0.27
L‐Lysine HCl	0.28	0.25	0.25	0.17	0.15	0.13	0.15	0.14	0.12
L‐Threonine	0.1	0.1	0.1	—	—	—	—	0.04	0.23
Common salt	0.24	0.25	0.26	0.26	0.27	0.29	0.29	0.30	0.31
**Calculated analysis (%)**									
Metabolisable energy (kcal/kg)	2925	3025	3125	3050	3150	3250	3100	3200	3300
Crude protein	21.96	22.72	23.47	20.33	21.0	21.66	18.40	19.0	19.59
Calcium	0.96	0.99	1.03	0.85	0.89	0.92	0.82	0.84	0.87
Available P	0.38	0.39	0.40	0.47	0.49	0.50	0.33	0.34	0.35
Methionine	0.71	0.73	0.77	0.65	0.67	0.70	0.41	0.56	0.60
Met + Cys	1.02	1.05	1.01	0.93	0.96	1.0	0.67	0.83	0.90
Lysine	1.28	1.33	1.38	1.03	1.05	1.09	1.03	1.06	1.69
Threonine	0.99	1.02	1.04	0.87	0.88	0.90	0.76	0.82	1.02

^a^ Low energy: ME, 3% less than the recommended level; recommended (rec) energy: ME, as per the guidelines for the Ross 308 strain, 2019; high energy: ME, 3% more than the recommended level.

^b^ Vitamin premix provided per kg of diet: vitamin A (retinyl acetate), 15,000 U; vitamin D35,00000 U; vitamin E (DL‐α‐tocopheryl acetate), 80 mg; vitamin K, 5 mg; thiamin, 3 mgriboflavin,1010 mg; pyridoxine, 5 mg; vitamin B_12_, 0.02 mg; niacin, 70 mg; choline chloride1,80000 mg; folic acid, 2 mg; biotin, 0.4 mg; pantothenic acid, 20 mg.

^c^ Mineral premix provided per kg of diet: Mn (manganese sulfate), 100 mg; Zn (zinc sulfate), 65 mg; Cu (copper sulfate), 5 mg; Se (sodium selenite), 0.22 mg; I (calcium iodate), 0.5 mg; and cobalt, 0.5 mg.

### 2.2 Growth Performance

Broilers were weighed on Days 10, 24, and 42 following a 3‐h feed deprivation period to assess average body weight (BW) per pen, from which WG was calculated. FI was recorded from Day 1 to 10 (starter phase), from Day 11 to 24 (grower phase), and from Day 25 to 42 (finisher phase). Average daily FI and FCR (expressed as g of feed per g of gain) were calculated.

### 2.3 Blood Metabolites

On Day 42, one bird was randomly selected from each pen, and 4 mL blood samples was collected from the brachial vein using a sterile syringe. The samples were transferred to tubes without anticoagulants and centrifuged at 2000 *g* for 10 min. Serum samples were collected and stored at −20°C until laboratory analysis. Appropriate laboratory kits (Ziestchem kit, Zist Shimi Company) were used to determine low‐density lipoproteins (LDL‐C), high‐density lipoproteins (HDL‐C), triglycerides (TG), and total cholesterol (Chol) concentrations following the manufacturer's instructions (Aziz‐Aliabadi et al. 2024).

### 2.4 Digestibility Assay

Apparent total tract digestibility measurements were conducted from Days 18 to 22 by adding 3 g/kg of chromium oxide (Cr_2_O_3_) to the diet as a non‐digestible marker. Excreta were collected from each pen several times daily during the period from Days 20 to 22. To enhance sampling accuracy, one‐third of the surface area of each pen was covered with a thin wooden board throughout the three‐day collection period. Excreta deposited on the board were collected multiple times daily. After removing contaminants such as feathers and bedding, samples were temporarily stored in a refrigerator at 4°C. At the end of the collection period, all samples from each pen were thoroughly mixed. Following homogenization, a 200 g sample per replicate was collected and stored at −20°C for further analysis. Feed and excreta samples were dried at 55°C for 72 h, ground to pass through a 0.5‐mm sieve, and stored at 22°C in airtight containers. All samples were analyzed for the marker using the method described by Fenton and Fenton (1979) and for CP (method 2001.11), lipids (method 920.39), and ash (method 942.05) according to standard methods of the Association of Official Analytical Chemists (AOAC 2005). The apparent total tract digestibility of CP, lipids, and ash was calculated using the following equation:

$$\text{TTAD}\;\left(\%\right)=100-100\times \left(\frac{\%\text{chromium in feed}}{\%\text{chromium in excreta}}\times \frac{\%\text{nutrient in excreta}}{\%\text{nutrient in feed}}\right)$$

### 2.5 Carcass Traits

At 42 days of age, one bird per replicate, selected based on proximity to the average weight of the replicate, was euthanized. Carcass weight was measured after the removal of blood, feathers, head, feet, abdominal fat pad, and all viscera. Carcass yield, as well as the weights of thighs, wings, and breast, was calculated as percentages relative to live BW. Additionally, the relative weights of various internal organs, including liver, heart, spleen, gizzard, bursa of Fabricius, and abdominal fat, were expressed as a percentage of live BW.

### 2.6 Statistical Analysis

All data were assessed for normality using the univariate procedure of SAS prior to statistical analysis. Subsequently, the data were analyzed using the General Linear Model (GLM) procedure of SAS, version 9.4 (SAS Institute 2012), in a completely randomized design structured as a 3 × 3 factorial arrangement (three SDs of 10, 13, or 16 birds/m^2^ in floor pens and three dietary ME levels). Mean comparisons were performed using Duncan's multiple range test at a significance level of 5%.

## 3 Results

### 3.1 Growth Performance

The effects of varying dietary ME levels, SD, and their interaction on FI in broiler chickens are summarized in Table 2. In the starter phase, FI was influenced by both dietary ME levels and SD, as well as their interaction. Notably, an increase in dietary ME resulted in a significant decrease in FI (*p* < 0.05). The lowest FI was observed in chickens fed with high‐energy diets, which, while not significantly different from the recommended‐energy treatment, was significantly lower than that of the low‐energy group. Conversely, an increase in SD during the starter phase led to a significant increase in FI (*p* < 0.01). Birds raised at a density of 16 birds/m^2^ (34.5 kg final BW/m^2^) exhibited significantly higher FI compared to those at densities of 10 and 13 birds/m^2^ (22.2 and 28.1 kg final BW/m^2^, respectively). There was also a significant interaction effect between SD and dietary ME on FI during the starter phase (*p* < 0.01). The highest FI was observed in chicks fed low‐energy diets at a density of 16 birds/m^2^, while those fed high‐energy diets at a density of 10 birds/m^2^ had the lowest FI. In the grower, finisher, and overall rearing periods, FI was not significantly affected by dietary ME, SD, or their interaction (*p* > 0.05).

**TABLE 2:** Effect of dietary ME level and stocking density on growth performance of broiler chicks from 1 dayso 42 day of age.

Treatments	Feed intake (g/bird/day)				Weight gain (g/bird/day)				Feed conversion ratio			
	1–10 day	11–24 day	25–42 day	1–42 day	1–10 day	11–24 day	25–42 day	1–42 day	1–10 day	11–24 day	25–42 day	1–42 day
ME												
Low	23.1^a^	73.8	145.2	92.3	14.7	42.4	70.7^b^	48.4^b^	1.57^a^	1.74^a^	2.03^a^	1.91^a^
Rec	22.6^ab^	74.8	149.6	94.4	15.1	44.6	73.7^ab^	52.5^a^	1.50^b^	1.67^b^	1.88^b^	1.79^b^
High	22.3^b^	72.7	147.4	92.7	15.0	44.0	79.0^a^	52.1^a^	1.49^b^	1.65^b^	1.86^b^	1.78^b^
SEM	0.16	0.64	1.11	0.57	0.13	0.47	0.94	0.49	0.01	0.02	0.02	0.01
SD^1^ (kg final BW/m^2^)												
10 (22.2)	22.3^b^	74.1	150.9	94.7	14.5^b^	43.7	79.3	52.0	1.53	1.70	1.90	1.82
13 (28.1)	22.4^b^	73.8	145.8	92.4	14.8^ab^	44.3	75.4	50.6	1.51	1.67	1.94	1.83
16 (34.5)	23.3^a^	73.3	145.5	92.3	15.4^a^	43.1	75.4	50.4	1.51	1.70	1.93	1.83
SEM	0.16	0.64	1.11	0.57	0.13	0.47	0.94	0.49	0.01	0.02	0.02	0.01
ME × SD^1^ (kg final BW/m^2^)												
Low												
10 (21.8)	23.0^ab^	75.2	150.5	95.0	14.8^ab^	43.1	77.0^ab^	50.9^abc^	1.55^ab^	1.75	1.95^abc^	1.86^ab^
13 (26.4)	22.5^ab^	73.7	143.9	91.6	14.2^b^	43.5	69.0^b^	47.4^bc^	1.58^a^	1.69	2.09^a^	1.93^a^
16 (32.1)	23.8^a^	72.4	141.3	90.3	15.0^ab^	40.7	69.2^b^	46.8^c^	1.58^a^	1.78	2.04^ab^	1.93^a^
Rec												
10 (22.2)	22.2^ab^	75.7	152.5	95.9	14.1^b^	44.0	79.1^a^	51.9^ab^	1.58^ab^	1.72	1.92^bc^	1.84^abc^
13 (29.6)	21.8^b^	74.9	147.6	93.4	15.2^ab^	46.1	80.1^a^	53.3^a^	1.44^b^	1.63	1.84^c^	1.75^bc^
16 (36.4)	23.7^a^	73.7	148.6	93.9	15.9^a^	43.8	79.0^a^	53.2^ab^	1.49^ab^	1.68	1.88^c^	1.79^bc^
High												
10 (22.8)	21.7^b^	71.5	149.6	93.1	14.7^ab^	44.1	81.8^a^	53.3^a^	1.48^ab^	1.62	1.83^c^	1.75^c^
13 (28.4)	22.8^ab^	72.8	145.8	92.2	15.1^ab^	43.1	75.5^ab^	51.0^abc^	1.51^ab^	1.69	1.89^bc^	1.8^bc^
16 (35.7)	22.4^ab^	73.8	145.7	92.8	15.2^ab^	45.0	78.1^ab^	52.1^ab^	1.47^ab^	1.64	1.88^c^	1.78^bc^
SEM	0.28	1.11	1.92	0.99	0.23	0.81	1.63	0.85	0.02	0.03	0.03	0.02
*p* value												
ME	0.03	0.48	0.30	0.31	0.31	0.14	< 0.01	< 0.01	< 0.01	0.04	< 0.01	< 0.01
SD	< 0.01	0.88	0.10	0.18	0.01	0.61	0.04	0.131	0.55	0.71	0.34	0.74
ME × SD	< 0.01	0.88	0.40	0.47	0.02	0.34	< 0.01	< 0.01	< 0.01	0.30	< 0.01	< 0.01

*Note:* a–c Means with different superscripts within a column are different at *p* < 0.05. Low energy: ME, 3% less than the recommended level; recommended energy: ME, as per the guidelines for the Ross 308 strain, 2019; high energy: ME, 3% more than the recommended level.

Abbreviations: ME, metabolisable energy; Rec, recommended; SD, stocking density; SEM, standard error of means.

^1^ The numbers 10, 13, and 16 refer to stocking densities of 10, 13, and 16 birds/m^2^ floor pens.

The effect of dietary ME levels, SD, and their interaction on daily WG in broilers is detailed in Table 2. No significant differences in average daily WG were observed in the starter and grower phases (*p* > 0.05); however, significant differences were noted during the finisher phase and the entire experimental period (*p* < 0.01). Chicks fed high‐energy diets demonstrated significantly greater WG compared to those fed low‐energy diets, although no significant difference was found between the high and recommended‐energy groups (*p* < 0.01). The effect of SD on the average daily gain of broilers was also significant only in the starter phase (*p* < 0.05). Daily WG at a SD16 was higher than at a SD10 but was not different from that at a SD13. SD had no significant effect on daily WG during the growth, finishing, and entire rearing periods. The interaction effect of dietary ME and SD on the average daily WG of broilers during the growth period was not significant but was significant in the other three periods measured. The highest WG was observed in the starter phase at a SD16 and fed with the recommended‐energy level of the diet, in the finishing period at a SD10 and fed with a high‐energy level, and in the entire rearing period at a SD13 and fed with the recommended‐energy level of the diet (Table 2).

The effects of dietary ME levels, SD, and their interaction on FCR are presented in Table 2. Dietary ME significantly influenced FCR during the starter, grower, and finisher phases, as well as the entire rearing period (*p* < 0.01). In these periods, FCR did not differ significantly between chickens fed high and recommended‐energy diets; however, both groups exhibited a significantly better FCR compared to those fed low‐energy diets. SD did not significantly impact FCR across any rearing periods or the overall experimental period (*p* > 0.05). The interaction between SD and dietary ME on FCR was significant during other rearing periods and the entire experimental period, except in the grower phase (*p* < 0.05).

### 3.2 Blood Metabolites

The influence of dietary ME levels, SD, and their interaction on serum concentrations of total cholesterol, triglycerides, high‐density lipoprotein (HDL), and low‐density lipoprotein (LDL) in broiler chickens is shown in Table 3. Total cholesterol, triglycerides, and HDL concentrations were significantly affected by dietary ME levels (*p* < 0.05). Specifically, increasing dietary ME to the recommended or high level significantly elevated these metabolites compared to those fed low‐energy diets (*p* < 0.05). LDL concentration was unaffected by dietary ME levels. SD did not significantly affect any of the serum factors measured. Significant interaction effects between dietary ME and SD were observed for serum cholesterol and triglyceride concentrations (*p* < 0.05), while no significant effects were noted for HDL and LDL concentrations. The highest total cholesterol concentration was found in the blood of chickens fed a high‐energy diet at SD16, while the lowest concentration was observed in the blood of those fed a low‐energy diet at SD13. For triglycerides, the highest concentration was detected in chickens fed high‐energy diets at SD10, and the lowest in those fed a low‐energy diet at SD13.

**TABLE 3:** Effect of dietary ME level and stocking density on blood metabolites (42 day of age) and apparent total tract digestibility (20 to 22 day of age) in broiler chicks.

Treatments		Blood metabolites, mg/dL				Apparent total tract digestibility, %		
		Chol	HDL‐C	LDL‐C	TG	CP	Lipid	Ash
ME								
Low		97.3^b^	53.3^b^	16.9	75.1^b^	84.6	89.7^b^	57.4
Rec		103.1^a^	61.7^a^	17.3	97.9^a^	83.4	92.4^ab^	56.5
High		106.7^a^	58.3^ab^	16.6	107.3^a^	83.9	93.5^a^	56.8
SEM		1.05	1.47	0.65	3.18	0.43	0.57	0.80
SD^1^ (kg final BW/m^2^)								
10 (22.2)		104.5	60.0	18.4	94.8	83.32	91.3	56.8
13 (28.1)		100.2	59.4	16.2	93.4	83.6	92.4	57.9
16 (34.5)		102.5	53.6	16.2	92.2	84.9	91.8	55.9
SEM		1.05	1.47	0.65	3.18	0.43	0.57	0.80
ME × SD^1^ (kg final BW/m^2^)								
Low								
10	21.8	99.2^ab^	53.5	21.1	77.1^ab^	84.6^ab^	88.3	55.9
13	26.4	94.4^b^	53.1	13.7	71.6^c^	85.4^ab^	90.7	61.6
16	32.1	98.3^ab^	53.2	16.0	76.5^bc^	83.7^ab^	90.0	54.7
Rec								
10	22.2	105.0^ab^	66.5	18.3	95.2^abc^	83.3^ab^	93.0	57.7
13	29.6	102.1^ab^	65.1	18.8	100.9^abc^	83.7^ab^	92.8	56.1
16	36.4	102.4^ab^	53.8	14.8	97.7^abc^	83.0^b^	91.3	55.6
High								
10	22.8	109.2^a^	60.2	15.9	112.0^a^	82.0^b^	92.7	56.9
13	28.4	104.2^ab^	60.2	16.2	107.6^a^	81.7^b^	93.7	56.1
16	35.7	106.8^a^	53.9	17.7	102.4^ab^	88.0^a^	94.1	57.5
SEM		1.82	2.55	1.13	5.51	0.74	0.99	1.39
*p* value								
ME		< 0.01	0.05	0.88	< 0.01	0.29	0.03	0.91
Stocking density		0.11	0.19	0.23	0.86	0.12	0.74	0.64
ME × SD		< 0.01	0.14	0.19	< 0.01	< 0.01	0.28	0.76

*Note:* a–c Means with different superscripts within a column are different at *p* < 0.05. Low energy: ME, 3% less than the recommended level; recommended energy: ME, as per the guidelines for the Ross 308 strain, 2019; high energy: ME, 3% more than the recommended level.

Abbreviations: Chol, total cholesterol; CP, crude protein; HDL‐C, high density lipoprotein cholesterol; LDL‐C, low density lipoprotein cholesterol; ME, metabolizable energy; Rec, recommended; SD, stocking density; SEM, standard error of means; TG, triglycerides.

^1^ The numbers 10, 13, and 16 refer to stocking densities of 10, 13, and 16 birds/m^2^ floor pens.

### 3.3 Digestibility Assay

The effects of dietary ME levels, SD, and their interaction on the ATTD of a number of nutrients are reported in Table 3. Dietary ME levels did not significantly affect the ATTD of CP and ash; however, lipid digestibility was significantly influenced (*p* < 0.05). Lipid digestibility increased with higher dietary ME levels, with significantly greater digestibility observed in chickens fed a high‐energy diet compared to those fed a low‐energy diet. SD had no significant effect on the digestibility of CP, lipids, or ash (*p* > 0.05). The interaction of dietary ME and SD significantly affected protein digestibility but did not impact lipid or ash digestibility. The highest protein digestibility was obtained in the group fed a high‐energy diet at a density of 16 birds/m^2^.

### 3.4 Carcass Traits

Results for carcass traits and relative internal organ weights of broilers fed diets with varying ME levels at different SDs at 42 days of age are presented in Table 4. Carcass and viscera weights are expressed as percentages of the live weight of the birds at slaughter. Neither dietary ME levels nor SD, nor their interaction, had a significant effect on the percentage of peeled carcass, carcass components, or relative viscera weight (*p* > 0.05).

**TABLE 4:** Effect of dietary ME level and stocking density on carcass cuts and internal organs of broiler chicks at 42 day of age.

Treatments		% of live weigh											
		Breast	Tights	Neck+ back	Wings	Heart	Liver	Gizzard	Bursa of Fabricius	Spleen	Pancreas	Abdominal fat	Edible carcass^a^
ME													
Low		23.0	19.0	14.1	5.45	0.53	2.22	1.47	0.054	0.12	0.29	1.60	61.6
Rec		24.4	18.2	14.3	5.15	0.56	2.14	1.39	0.050	0.10	0.28	1.52	62.1
High		25.0	18.4	13.8	5.15	0.51	2.17	1.37	0.050	0.11	0.28	1.39	62.4
SEM		0.33	0.24	0.25	0.08	0.01	0.05	0.04	0.004	0.01	0.01	0.06	0.40
SD^b^ (kg final BW/m^2^)													
10 (22.2)		24.2	19.2	14.3	5.27	0.51	2.09	1.40	0.05	0.11	0.28	1.60	63.0
13 (28.1)		24.7	18.2	13.3	5.37	0.54	2.20	1.41	0.05	0.11	0.27	1.35	61.6
16 (34.5)		23.5	18.2	14.6	5.12	0.55	2.24	1.43	0.05	0.10	0.29	1.55	61.4
SEM		0.33	0.24	0.25	0.08	0.01	0.05	0.04	0.004	0.01	0.01	0.06	0.40
ME× SD^b^ (kg final BW/m^2^)													
Low													
10	21.8	23.1	19.6	14.3	5.37	0.45	2.17	1.58	0.05	0.09	0.28	1.59	62.4
13	26.4	23.8	19.1	13.2	5.59	0.58	2.12	1.33	0.06	0.13	0.26	1.43	61.7
16	32.1	22.1	18.3	14.7	5.41	0.55	2.37	1.51	0.05	0.12	0.30	1.77	60.5
Rec													
10	22.2	24.0	19.0	14.9	5.41	0.58	2.10	1.27	0.05	0.12	0.27	1.57	63.2
13	29.6	25.2	17.7	12.9	5.15	0.55	2.22	1.53	0.05	0.10	0.30	1.43	60.9
16	36.4	24.1	18.1	15.0	4.90	0.54	2.09	1.38	0.05	0.09	0.27	1.55	62.1
High													
10	22.8	25.6	18.9	13.6	5.05	0.49	2.00	1.34	0.05	0.10	0.28	1.66	63.2
13	28.4	25.0	18.0	13.9	5.37	0.51	2.25	1.38	0.05	0.12	0.26	1.39	62.2
16	35.7	25.0	18.3	13.9	5.04	0.55	2.26	1.40	0.05	0.09	0.29	1.32	61.5
SEM		0.57	0.42	0.43	0.14	0.02	0.09	0.07	0.004	0.02	0.02	0.10	0.69
*p* value													
ME		0.05	0.45	0.77	0.23	0.44	0.82	0.61	0.76	0.48	0.96	0.30	0.74
Stocking density		0.31	0.23	0.14	0.43	0.42	0.51	0.96	0.89	0.29	0.80	0.15	0.30
ME × SD		0.27	0.67	0.48	0.51	0.44	0.85	0.71	0.97	0.17	0.96	0.34	0.84

*Note:* Low energy: ME, 3% less than the recommended level; recommended energy: ME, as per the guidelines for the Ross 308 strain, 2019; high energy: ME, 3% more than the recommended level.

Abbreviations: ME, metabolisable energy; Rec, recommended; SD, stocking density; SEM, standard error of means.

^a^ Carcass as peeled.

^b^ The numbers 10, 13 and 16 refer to stocking densities of 10, 13 and 16 birds/m^2^ floor pens.

## 4 Discussion

### 4.1 Growth Performance

The present study investigated the effects of varying SDs and dietary ME levels on growth performance, blood metabolites, nutrient digestibility, and carcass traits in Ross 308 broiler chicks. Our findings indicated that both stocking density and dietary ME levels significantly influence growth performance indices, nutrient digestibility, and serum metabolite concentrations, although the effects on carcass traits appear less pronounced.

The observed increase in FI with higher stocking density during the starter phase aligns with previous findings, suggesting that competition for resources can stimulate increased FI among broilers (Stamp Dawkins et al. 2004). The significant reduction in FI associated with high‐energy diets, particularly in the starter phase, may be attributed to the increased caloric density of these diets, leading to satiety at lower volumes of feed (Classen 2017). Our results regarding FI both confirm and contrast with previous reports. For instance, Hosseini‐Vashan et al. (2010) reported that different dietary ME levels with a constant energy‐to‐protein ratio significantly affected FI during the starter phase; however, they found no effects during the grower and finisher phases, attributing this to the physical limitations of the digestive tract. Similarly, Azizi et al. (2011) observed that FI was not affected by dietary ME levels during specific periods. Zhou et al. (2024) reported no effect of increased stocking density on FI, attributing this to adequate feeder space and reduced distances to feeders. Conversely, Dozier 3rd et al. (2005) stated that SD has a significant effect on FI, so that it decreases with increasing SD. Also, Abudabos, Abdelrahman, et al. (2013) Abudabos, Samara, Hussein, Al‐Ghadi, et al. (2013) concluded that increasing stocking density from 28 to 40 kg body weight per m^2^ had obvious adverse effects on broiler performance and could compromise their welfare.

In the current study, the higher FI in the starter phase at SD16 compared to the other two SDs, which resulted in a significant increase in daily WG, may be due to greater competition among chicks and higher FI, while they did not suffer from space constraints at this age.

While growth rates did not significantly differ during the starter and grower phases, the enhanced WG observed in the finisher phase for birds on high‐energy diets underscores the importance of ME availability during critical growth periods. Although Azizi et al. (2011) reported that dilution of energy and protein in the diet had no significant effect on WG, Kamran et al. (2008) noted a significant increase in weight for chicks fed high‐energy and protein diets during the finisher phase and overall rearing period. Contrarily, Lee et al. (2024) indicated that the effect of dietary ME on body weight in early ages was significant, with increased body weight correlating with higher dietary ME levels. Li et al. (2019) found that stocking density significantly affected body WG, while Guinebretiere et al. (2024) reported reduced WG at higher densities, though the differences were not significant.

The FCR results indicated that dietary ME levels play a crucial role in feed efficiency, as supported by literature demonstrating improved FCR with optimized dietary ME. The lack of significant impact from stocking density on FCR suggests that, within the tested density range, dietary ME had a more dominant influence on feed efficiency. This reflects the complex interactions between environmental conditions and dietary formulations in broiler management. In the present study, as observed from the effects of dietary ME and SD on FCR, the role of SD on FCR was very weak and the best FCR was observed in treatments fed with high levels of dietary ME. In agreement with the current study, Lee et al. (2024) found that high‐energy diets significantly improve FCR of broilers. Similarly, Li et al. (2019) reported that stocking density had no significant effect on FCR. While the lack of significant effect of high SDs on FCR in this experiment is not clear to the authors, it has been stated that chronic stress, when not severe, can lead to adaptation without negatively impacting performance. Some researchers have reported conflicting results; Kamran et al. (2008) found that FCR was unaffected by dietary ME content in the starter phase, while Bilgili and Hess (1995) noted that FCR decreases with increasing stocking density due to reduced movement space, which lowers energy requirements for maintenance.

### 4.2 Blood Metabolites

Analysis of blood metabolites revealed that total cholesterol, triglycerides, and HDL levels were significantly influenced by dietary ME levels, with the highest concentrations observed in birds fed high‐energy diets. This aligns with the hypothesis that increased dietary ME can elevate lipid profiles (Viveros et al. 2011). However, LDL concentrations remained unaffected by dietary ME levels, indicating a potential ceiling effect or a compensatory mechanism in lipid metabolism that warrants further investigation. The lack of significant variation in serum factors with changing SDs suggests that environmental stressors related to space may not have been sufficient to elicit measurable changes in metabolic profiles under the conditions of this study. In the current study, the increased oil content of the diets with higher ME levels was expected to elevate blood cholesterol and triglyceride levels. The observed increase in HDL can be justified by the use of soybean oil, which contains unsaturated fatty acids. This finding is consistent with Stamp Dawkins et al. (2004), who also found no increase in cholesterol levels with increasing stocking density. It has been reported that increased dietary fat elevates blood HDL concentration, and higher levels of fat in broiler diets significantly increased blood cholesterol compared to lower fat levels (Ai et al. 2025).

### 4.3 Digestibility Assay

The digestibility assay results indicated that while lipid digestibility improved with higher ME diets, protein and ash digestibility remained unaffected. The significant interaction effect observed for CP digestibility may suggest that the combination of high‐energy diets and higher SDs could enhance nutrient uptake efficiency. This reflects the need for further exploration into feed formulations that optimize nutrient utilization under varying density conditions. Dietary fat slows the rate of food passage through the digestive tract, resulting in improved digestion and absorption (Svihus and Itani 2019).

### 4.4 Carcass Traits

Regarding carcass traits, the absence of significant differences in carcass yield and internal organ weights across dietary and density treatments suggests that, although growth performance parameters were influenced, overall carcass quality remained relatively stable. This finding indicates that broilers can be raised at varying SDs and ME levels without adversely affecting carcass traits, which is crucial for commercial production considerations.

In conclusion, our study highlights the critical role of dietary ME levels in influencing growth performance and metabolic health in broiler chicks, while stocking density impacts certain parameters primarily during the early life stages. These findings provide valuable insights for poultry nutritionists and producers in optimizing broiler management strategies to enhance productivity while ensuring animal welfare. Future research aimed at elucidating the underlying mechanisms governing these interactions could further refine dietary recommendations and housing strategies in commercial broiler production. In agreement with our findings, Kamran et al. (2008) found that dietary ME level had no significant effect on carcass characteristics of broilers. Ko et al. (2023) reported that dietary ME level with a fixed nutrient‐to‐energy ratio had no significant effect on live weight and carcass components. There is ongoing debate regarding the effects of dietary ME levels on carcass composition and quality, particularly concerning abdominal fat. In general, as long as the energy‐to‐protein ratio is maintained, the percentage of carcass fat remains stable; carcass fat tends to increase only when dietary ME increases independently (Ko et al. 2023). Additionally, Rahbari et al. (2025) found no significant effects of stocking density on the weights of carcass and internal organs such as liver, abdominal fat, and heart. The lack of significant effects on the weight of the bursa of Fabricius, a suitable indicator of stress, suggests that the stocking density in this experiment did not significantly impact chick stress levels. Ravindran et al. (2006) concluded that bursa weight decreases with increasing stocking density. The findings of Dozier 3rd et al. (2005) also indicated that varying SDs do not significantly affect abdominal fat weight. Given that increasing the stocking density to 16 birds/m^2^ did not negatively impact broiler performance, future studies may explore even higher densities.

## 5 Conclusions

Our findings indicated that high and recommended energy and nutrients of the diet improved growth performance and total tract lipid digestibility and increased cholesterol, HDL‐C, and triglyceride concentrations in the blood of broiler chicks without affecting blood LDL‐C. Stocking density had no remarkable effect on growth performance, blood factors, nutrient digestibility, and carcass traits. However, as stocking density increased from 13 to 16, feed intake and weight gain of chicks increased in the starter phase, which may be due to learning from each other or competition for feed consumption at early ages. Therefore, considering other management conditions such as house ventilation and desired broiler slaughter weight, any of the three stocking densities of 10, 13, or 16 birds/m^2^ along with recommended or high‐energy diets can be considered for breeding.

## Author Contributions

**Zeyad Kamal Imari:** data curation, writing – original draft, writing – review and editing. **Saeid Jahandost Ardin:** investigation, data curation (equal), writing – review and editing (equal). **Ahmad Hassanabadi:** project administration; methodology; conceptualization; funding acquisition; writing – review and editing (lead).

## Conflicts of Interest

The authors declare no conflicts of interest.

## Data Availability

The data that support the findings of this study are available from the corresponding author upon reasonable request.

## References

[fsn370836-bib-0001] Abudabos, A. M. , M. M. Abdelrahman , H. M. Yehia , M. Q. Al‐Ghadi , and I. A. Alhidary . 2013. “Effect of Stocking Density on Intestinal Histology and Ileal Bacterial Count in Broilers.” Asian Journal of Animal and Veterinary Advances 8: 740–746. 10.3923/ajava.2013.740.746.

[fsn370836-bib-0002] Abudabos, A. M. , E. Samara , E. O. Hussein , R. M. Al‐Atiyat , and A. Al‐Haidary . 2013. “Influence of Stocking Density on Welfare Indices of Broilers.” Italian Journal of Animal Science 12, no. 2: e35. 10.4081/ijas.2013.e35.

[fsn370836-bib-0003] Abudabos, A. M. , E. M. Samara , E. O. Hussein , M. A. Q. Al‐Ghadi , and R. M. Al‐Atiyat . 2013. “Impacts of Stocking Density on the Performance and Welfare of Broiler Chickens.” Italian Journal of Animal Science 12, no. 1: e11. 10.4081/ijas.2013.e11.

[fsn370836-bib-0004] Ai, H. , Y. Y. Lee , Y. Lu , et al. 2025. “Effect of Structured Lipids as Dietary Supplements on the Fatty Acid Profile, Carcass Yield, Blood Chemistry, and Abdominal Fat Deposition of Female Broilers.” Poultry Science 104: 104579. 10.1016/j.psj.2024.104579. PMC11681861 39657466

[fsn370836-bib-0005] AOAC International . 2005. Official Methods of Analysis. 18th ed. AOAC Int.

[fsn370836-bib-0006] Astaneh, I. Y. , M. Chamani , S. N. Mousavi , A. A. Sadeghi , and M. A. Afshar . 2018. “Effects of Stocking Density on Performance and Immunity in Ross 308 Broiler Chickens.” Kafkas Üniversitesi Veteriner Fakültesi Dergisi 24: 483–489. 10.9775/kvfd.2017.18869.

[fsn370836-bib-0007] Aviagen . 2019. Ross 308 Broiler: Nutrition Specifications. Aviagen Limited.

[fsn370836-bib-0008] Aziz‐Aliabadi, F. , F. Amirzadeh‐Garou , A. Hassanabadi , and H. Noruzi . 2024. “Investigating the Effect of Sesame Meal Replacement for Soybean Meal in Diets With Different Levels of Calcium and Phytase Enzyme in Broiler Chickens.” Veterinary Medicine and Science 10: e1379. 10.1002/vms3.1379. 38358071 PMC10867867

[fsn370836-bib-0009] Azizi, B. , G. Sadeghi , A. Karimi , and F. Abed . 2011. “Effects of Dietary Energy and Protein Dilution and Time of Feed Replacement From Starter to Grower on Broiler Chickens Performance.” Journal of Central European Agriculture 12: 44–52. 10.5513/JCEA01/12.1.879.

[fsn370836-bib-0010] Bar, A. , M. Shani , C. S. Fullmer , M. E. Brindak , and S. Striem . 1990. “Modulation of Chick Intestinal and Renal Calbindin Gene Expression by Dietary Vitamin D3, 1, 25‐Dihydroxyvitamin D3, Calcium and Phosphorus.” Molecular and Cellular Endocrinology 72: 23–31. 10.1016/0303-7207(90)90236-2. 2177015

[fsn370836-bib-0011] Bilgili, S. F. , and J. B. Hess . 1995. “Placement Density Influences Broiler Carcass Grade and Meat Yields.” Journal of Applied Poultry Research 4: 384–389. 10.1093/japr/4.4.384.

[fsn370836-bib-0012] Buijs, S. , E. Van Poucke , S. Van Dongen , L. Lens , J. Baert , and F. A. M. Tuyttens . 2012. “The Influence of Stocking Density on Broiler Chicken Bone Quality and Fluctuating Asymmetry.” Poultry Science 91: 1759–1767. 10.3382/ps.2011-01859. 22802165

[fsn370836-bib-0013] Classen, H. L. 2017. “Diet Energy and Feed Intake in Chickens.” Animal Feed Science and Technology 233: 13–21. 10.1016/j.anifeedsci.2016.03.004.

[fsn370836-bib-0014] Davami, A. , M. J. Wineland , W. T. Jones , R. L. Ilardi , and R. A. Peterson . 1987. “Effects of Population Size, Floor Space, and Feeder Space Upon Productive Performance, External Appearance, and Plasma Corticosterone Concentration of Laying Hens.” Poultry Science 66: 251–257. 10.3382/ps.0660251. 3588491

[fsn370836-bib-0015] Dohms, J. E. , and A. Metz . 1991. “Stress—Mechanisms of Immunosuppression.” Veterinary Immunology and Immunopathology 30: 89–109. 10.1016/0165-2427(91)90011-Z. 1781159

[fsn370836-bib-0016] Dozier, W. A., 3rd , J. P. Thaxton , S. L. Branton , et al. 2005. “Stocking Density Effects on Growth Performance and Processing Yields of Heavy Broilers.” Poultry Science 84: 1332–1338. 10.1093/ps/84.8.1332. 16156220

[fsn370836-bib-0017] Fenton, T. W. , and M. Fenton . 1979. “An Improved Procedure for the Determination of Chromic Oxide in Feed and Feces.” Canadian Journal of Animal Science 59: 631–634. 10.4141/cjas79-08.

[fsn370836-bib-0018] Guinebretiere, M. , L. Warin , J. P. Moysan , et al. 2024. “Effects of Strain and Stocking Density on Leg Health, Activity, and Use of Enrichments in Conventional Broiler Chicken Production.” Poultry Science 103: 103993. 10.1016/j.psj.2024.103993. PMC11298928 39002370

[fsn370836-bib-0019] Gursu, M. F. , M. Onderci , F. Gulcu , and K. Sahin . 2004. “Effects of Vitamin C and Folic Acid Supplementation on Serum Paraoxonase Activity and Metabolites Induced by Heat Stress In Vivo.” Nutrition Research 24: 157–164. 10.1016/j.nutres.2003.11.008.

[fsn370836-bib-0020] Hosseini‐Vashan, S. J. , A. R. Jafari‐Sayadi , A. Golian , G. Motaghinia , M. Namvari , and M. Hamedi . 2010. “Comparison of Growth Performance and Carcass Characteristics of Broiler Chickens Fed Diets With Various Energy and Constant Energy to Protein Ratio.” Journal of Animal and Veterinary Advances 9: 2565–2570. 10.3923/javaa.2010.2565.2570.

[fsn370836-bib-0021] Kamran, Z. , M. Sarwar , M. Nisa , et al. 2008. “Effect of Low‐Protein Diets Having Constant Energy‐To‐Protein Ratio on Performance and Carcass Characteristics of Broiler Chickens From One to Thirty‐Five Days of Age.” Poultry Science 87: 468–474. 10.3382/ps.2007-00180. 18281572

[fsn370836-bib-0044] Ko, H. , J. Wang , J. W. C. Chiu , and W. K. Kim . 2023. “Effects of Metabolizable Energy and Emulsifier Supplementation on Growth Performance, Nutrient Digestibility, Body Composition, and Carcass Yield in Broilers.” Poultry Science 102: 102509. 10.1016/j.psj.2023.102509. PMC9932563 36745956

[fsn370836-bib-0022] Lee, W. D. , H. J. Kim , H. S. Kim , et al. 2024. “Dietary Energy Levels Affect Productivity, Meat Quality, Blood Variables, Energy Efficiency and Welfare Indicators in Broilers Under Welfare Rearing Conditions.” Italian Journal of Animal Science 23: 1325–1335. 10.1080/1828051X.2024.2398175.

[fsn370836-bib-0023] Li, X. M. , M. H. Zhang , S. M. Liu , et al. 2019. “Effects of Stocking Density on Growth Performance, Growth Regulatory Factors, and Endocrine Hormones in Broilers Under Appropriate Environments.” Poultry Science 98: 6611–6617. 10.3382/ps/pez505. PMC8913966 31504910

[fsn370836-bib-0024] Nahashon, S. N. , N. Adefope , A. Amenyenu , J. Tyus II , and D. Wright . 2009. “The Effect of Floor Density on Growth Performance and Carcass Characteristics of French Guinea Broilers.” Poultry Science 88: 2461–2467. 10.3382/ps.2008-00514. 19834101

[fsn370836-bib-0025] Najafi, P. , I. Zulkifli , N. Amat Jajuli , et al. 2015. “Environmental Temperature and Stocking Density Effects on Acute Phase Proteins, Heat Shock Protein 70, Circulating Corticosterone and Performance in Broiler Chickens.” International Journal of Biometeorology 59: 1577–1583. 10.1007/s00484-015-0964-3. 25649005

[fsn370836-bib-0026] Rahbari, S. , A. Salehi , S. D. Sharifi , and S. Honarbakhsh . 2025. “Dietary Omega‐3 Fatty Acids Affect the Growth Performance of Broiler Chickens Reared at High Stocking Density.” Poultry Science 104: 104468. 10.1016/j.psj.2024.104468. PMC11635653 39603183

[fsn370836-bib-0027] Ravindran, V. , D. V. Thomas , D. G. Thomas , and P. C. Morel . 2006. “Performance and Welfare of Broilers as Affected by Stocking Density and Zinc Bacitracin Supplementation.” Animal Science Journal 77: 110–116. 10.1111/j.1740-0929.2006.00327.x.

[fsn370836-bib-0028] SAS . 2012. SAS User's Guide: Statistics Version 9.4. SAS Institute. Inc.

[fsn370836-bib-0029] Shakeri, M. , I. Zulkifli , A. F. Soleimani , et al. 2014. “Response to Dietary Supplementation of L‐Glutamine and L‐Glutamate in Broiler Chickens Reared at Different Stocking Densities Under Hot, Humid Tropical Conditions.” Poultry Science 93: 2700–2708. 10.3382/ps.2014-03910. 25143595

[fsn370836-bib-0030] Simitzis, P. E. , E. Kalogeraki , M. Goliomytis , et al. 2012. “Impact of Stocking Density on Broiler Growth Performance, Meat Characteristics, Behavioural Components and Indicators of Physiological and Oxidative Stress.” British Poultry Science 53: 721–730. 10.1080/00071668.2012.745930. 23398415

[fsn370836-bib-0031] Singh, K. D. , P. S. Pramanik , S. Kumar , R. Kumar , A. K. Srivastav , and M. K. Verma . 2021. “Impact of Stocking Density on Gait Score and Mortality in Broiler Chickens.” Journal of Pharmaceutical Innovation 10: 210–214.

[fsn370836-bib-0032] Stamp Dawkins, M. , C. A. Donnelly , and T. A. Jones . 2004. “Chicken Welfare Is Influenced More by Housing Conditions Than by Stocking Density.” Nature 427: 342–344. 10.1038/nature02226. 14737165

[fsn370836-bib-0033] Sun, Z. W. , L. Yan , G. Yy , J. P. Zhao , H. Lin , and Y. M. Guo . 2013. “Increasing Dietary Vitamin D_3_ Improves the Walking Ability and Welfare Status of Broiler Chickens Reared at High Stocking Densities.” Poultry Science 92: 3071–3079. 10.3382/ps.2013-03278. 24235214

[fsn370836-bib-0034] Svihus, B. , and K. Itani . 2019. “Intestinal Passage and Its Relation to Digestive Processes.” Journal of Applied Poultry Research 28: 546–555. 10.3382/japr/pfy027.

[fsn370836-bib-0035] Swanson, J. C. 1995. “Farm Animal Well‐Being and Intensive Production Systems.” Journal of Animal Science 73: 2744–2751. 10.2527/1995.7392744x. 8582867

[fsn370836-bib-0036] Tsiouris, V. , I. Georgopoulou , C. Batzios , N. Pappaioannou , R. Ducatelle , and P. Fortomaris . 2015. “High Stocking Density as a Predisposing Factor for Necrotic Enteritis in Broiler Chicks.” Avian Pathology 44: 59–66. 10.1080/03079457.2014.1000820. 25563065

[fsn370836-bib-0037] Uzum, M. H. , and H. O. Toplu . 2013. “Effects of Stocking Density and Feed Restriction on Performance, Carcass, Meat Quality Characteristics and Some Stress Parameters in Broilers Under Heat Stress.” Revue de Médecine Vétérinaire 164: 546–554.

[fsn370836-bib-0038] Vanhonacker, F. , W. Verbeke , E. Van Poucke , S. Buijs , and F. A. Tuyttens . 2009. “Societal Concern Related to Stocking Density, Pen Size and Group Size in Farm Animal Production.” Livestock Science 123: 16–22. 10.1016/j.livsci.2008.09.023.

[fsn370836-bib-0039] Viveros, A. , S. Chamorro , M. Pizarro , I. Arija , C. Centeno , and A. Brenes . 2011. “Effects of Dietary Polyphenol‐Rich Grape Products on Intestinal Microflora and Gut Morphology in Broiler Chicks.” Poultry Science 90: 566–578. 10.3382/ps.2010-00889. 21325227

[fsn370836-bib-0040] Weeks, C. A. , and A. Butterworth , eds. 2004. Measuring and Auditing Broiler Welfare. CABI publishing.

[fsn370836-bib-0041] Wiseman, J. , and C. E. Lewis . 1998. “Influence of Dietary Energy and Nutrient Concentration on the Growth of Body Weight and of Carcass Components of Broiler Chickens.” Journal of Agricultural Science 131: 361–371. 10.1017/S0021859698005851.

[fsn370836-bib-0042] Zhang, Y. R. , L. S. Zhang , Z. Wang , et al. 2018. “Effects of Stocking Density on Growth Performance, Meat Quality and Tibia Development of Pekin Ducks.” Animal Science Journal 89: 925–930. 10.1111/asj.12997. 29682864

[fsn370836-bib-0043] Zhou, S. , P. Watcharaanantapong , X. Yang , et al. 2024. “Evaluating Broiler Welfare and Behavior as Affected by Growth Rate and Stocking Density.” Poultry Science 103: 103459. 10.1016/j.psj.2024.103459. PMC10847911 38308899

